# *LRP1* Repression by SNAIL Results in ECM Remodeling in Genetic Risk for Vascular Diseases

**DOI:** 10.1161/CIRCRESAHA.124.325269

**Published:** 2024-10-02

**Authors:** Lu Liu, Joséphine Henry, Yingwei Liu, Charlène Jouve, Jean-Sébastien Hulot, Adrien Georges, Nabila Bouatia-Naji

**Affiliations:** 1 Université Paris Cité, Inserm, PARCC, Paris, France.

**Keywords:** induced pluripotent stem cells, low density lipoprotein receptor-related protein-1, myocytes, smooth muscle, vascular diseases

## Abstract

**BACKGROUND:**

Genome-wide association studies implicate common genetic variations in the *LRP1* (low-density lipoprotein receptor-related protein 1 gene) locus at risk for multiple vascular diseases and traits. However, the underlying biological mechanisms are unknown.

**METHODS:**

Fine mapping analyses included Bayesian colocalization to identify the most likely causal variant. Human induced pluripotent stem cells were genome-edited using CRISPR-Cas9 (Clustered Regularly Interspaced Short Palindromic Repeats-CRISPR associated protein 9) to delete or modify candidate enhancer regions and generate *LRP1* knockout cell lines. Cells were differentiated into smooth muscle cells through a mesodermal lineage. Transcription regulation was assessed using luciferase reporter assay, transcription factor knockdown, and chromatin immunoprecipitation. Phenotype changes in cells were conducted using cellular assays, bulk RNA sequencing, and mass spectrometry.

**RESULTS:**

Multitrait colocalization analyses pointed at rs11172113 as the most likely causal variant in *LRP1* for fibromuscular dysplasia, migraine, pulse pressure, and spontaneous coronary artery dissection. We found the rs11172113-T allele to associate with higher *LRP1* expression. Genomic deletion in induced pluripotent stem cell–derived smooth muscle cells supported rs11172113 to locate in an enhancer region regulating *LRP1* expression. We found transcription factors MECP2 (methyl CpG binding protein 2) and SNAIL (Zinc Finger Protein SNAI1) to repress *LRP1* expression through an allele-specific mechanism, involving SNAIL interaction with disease risk allele. *LRP1* knockout decreased induced pluripotent stem cell–derived smooth muscle cell proliferation and migration. Differentially expressed genes were enriched for collagen-containing extracellular matrix and connective tissue development. *LRP1* knockout and deletion of rs11172113 enhancer showed potentiated canonical TGF-β (transforming growth factor beta) signaling through enhanced phosphorylation of SMAD2/3 (Mothers against decapentaplegic homolog 2/3). Analyses of the protein content of decellularized extracts indicated partial extracellular matrix remodeling involving enhanced secretion of CYR61 (cystein rich angiogenic protein 61), a known LRP1 ligand involved in vascular integrity and TIMP3 (Metalloproteinase inhibitor 3), implicated in extracellular matrix maintenance and also known to interact with LRP1.

**CONCLUSIONS:**

Our findings support allele-specific *LRP1* expression repression by the endothelial-to-mesenchymal transition regulator SNAIL. We propose decreased *LRP1* expression in smooth muscle cells to remodel the extracellular matrix enhanced by TGF-β as a potential mechanism of this pleiotropic locus for vascular diseases.

**Novelty and Significance**

**What Is Known?**

- Common genetic variants in *LRP1* (low-density lipoprotein receptor-related protein 1 gene), which encodes a multifunctional endocytic transmembrane protein, are associated with several vascular diseases identified by genome-wide association studies.
- While regulatory functions are suspected to be at play specifically in vascular smooth muscle cells (SMCs), the molecular mechanisms are unclear.

**What New Information Does This Article Contribute?**

- A single common variant intronic to *LRP1* (rs11172113) accounts for the genetic association with multiple arterial diseases, including migraine, fibromuscular dysplasia, spontaneous coronary artery dissection, and thoracic aortic dissection.
- rs11172113 is located in an enhancer that disrupts the action of a transcriptional repressor SNAIL (Zinc Finger Protein SNAI1) in SMCs derived from induced pluripotent stem cells.
- Lowering *LRP1* expression by deleting this enhancer or knocking out the gene in induced pluripotent stem cell–derived SMCs promotes canonical TGF-β (transforming growth factor beta) signaling and extracellular matrix remodeling,
- This study provides a molecular mechanism in a human cell model involving distant regulatory elements by a pleiotropic genetic locus and protein, with high relevance to the genetic risk for several vascular diseases.

Common genetic variants in *LRP1* were reported in genome-wide association studies to associate with several vascular diseases. LRP1 encodes an endocytic transmembrane protein with multiple biological roles and conflicting results about its role in vascular function in humans. Although it is suspected that regulatory functions in vascular SMCs are involved, the specific molecular mechanisms underlying these genetic associations are unknown. We demonstrate that one common variant (rs11172113) intronic to *LRP1* is the most likely causal variant at the association signals for migraine, fibromuscular dysplasia, spontaneous coronary artery dissection, and sporadic thoracic aortic dissection. We show that this variant resides in an enhancer region that interferes with the function of the transcriptional repressor SNAIL in SMCs derived from induced pluripotent stem cells. Reducing *LRP1* expression by either deleting this enhancer or knocking out the gene in the induced pluripotent stem cell–derived SMCs enhances canonical TGF-β signaling and promotes extracellular matrix remodeling. This research uncovers a molecular mechanism in a human cell model involving a distant regulatory element related to a pleiotropic genetic locus, highlighting its significance in the genetic risk for multiple vascular diseases.

**Meet the First Author, see p 1031**

Genome-wide association studies (GWAS) have established thousands of genomic locations in link with cardiovascular diseases.^1–4^ GWAS generally produces complex association signals that are difficult to transcribe into clear biological mechanisms. Identified risk loci in GWAS are often highly pleiotropic involving association signals related to several diseases and traits that may potentially share unknown molecular and cellular mechanisms.^5^ Causal variants at GWAS loci are difficult to determine due to high linkage disequilibrium between associated variants that result in the redundancy of statistical signals in most loci. In addition, associated variants are mostly noncoding^6,7^ and likely to be involved in tissue or cell type–specific gene expression regulation.^8^ The identification of causal variations and their biological significance is particularly challenging in the case of inaccessible tissues such as arteries.

Several GWASs have reported robust association signals on chromosome 12, in the first intron of the *LRP1* (low-density lipoprotein receptor-related protein 1) gene. First reported in migraine,^9^ *LRP1* was also described as a risk locus for sporadic thoracic aortic dissection^10^ and, more recently for fibromuscular dysplasia (FMD), an arteriopathy associated with resistant hypertension and stroke, and with an underdiagnosed form of ischemic heart disease named spontaneous coronary artery dissection (SCAD).^11,12^ *LRP1* locus is also associated with pulse pressure (PP),^4^ a cardiovascular risk factor indicative of arterial stiffness,^13^ and forced expiratory volume over vital capacity,^14^ a surrogate trait of obstructive lung disease.^15^ The molecular and cellular mechanisms underlying these genetic associations with this wide range of vascular diseases and traits are still to be determined.

LRP1 is an endocytic transmembrane protein member of the low-density lipoprotein receptor family that interacts with numerous biologically diverse ligands^16^ and was extensively studied in the context of several human diseases. LRP1 is involved in many cellular processes through its capacity to internalize a wide variety of extracellular proteins. LRP1 regulates the composition of the extracellular matrix (ECM) and interacts with key signaling pathways, such as TGF-β (transforming growth factor beta) or PDGF (platelet-derived growth factor) pathways.^17^ LRP1 is highly expressed in smooth muscle cells (SMCs) where it was demonstrated to maintain endocytosis and vascular integrity.^18^ LRP1 regulates SMCs proliferation, migration,^19^ and ECM deposition,^20^ in addition to controlling calcium efflux to maintain arterial contractility.^21^ Independent studies in mice and human cells suggested a role for SMC-expressed *LRP1* in atherosclerosis^19,22^ and pulmonary hypertension,^23^ with conflicting reports on the cellular outcome of LRP1 deficiency.^19,22,23^

In this integrative study, we report plausible regulatory mechanisms for the associations observed at the *LRP1* locus. We leveraged multiple trait associations at this highly pleiotropic locus to formally identify rs11172113 as the most likely causal variant for reported associations. We assessed the biological function of this variant using genetically modified SMCs derived from human induced pluripotent stem cells (iPSCs) through a mesoderm lineage. We confirmed rs11172113 to belong to an enhancer region intronic to *LRP1* and provide data supporting transcriptional repressors, SNAIL (Zinc Finger Protein SNAI1) and MECP2 (methyl CpG binding protein 2), to regulate *LRP1* expression in an allele-specific manner. We showed the binding of SNAIL to this enhancer was impaired by risk alleles for diseases. Finally, we found that cellular knockout of *LRP1* in iPSC-derived SMCs led to hyperactivation of TGF-β signaling and hypersecretion of TIMP3 (Metalloproteinase inhibitor 3) and CYR61 (cystein rich angiogenic protein 61), 2 LRP1 ligands involved in vascular integrity.

## Methods

### Data Availability

Supporting data are available within the article and Supplemental Material. Raw sequencing data are available on Sequence Read Archive (PRJNA1153322). Underlying data and details of statistical analyses are provided on the Open Science Framework.

### Genetic Analysis

Summary statistics were retrieved from individual studies.^11,14,24,25^ We used GCTA-COJO software (genome-wide complex trait analysis conditional and joint association analysis) to perform conditional analysis.^26^ The local linkage desequilibrium structure was provided in a reference file from the haplotype reference consortium (http://www.haplotype-reference-consortium.org/site). LocusZoom (http://locuszoom.org/) was used for visualization. Signal colocalization was evaluated using the R coloc package (v5.1.0) with default values as priors.^27^ Multitrait colocalization to prioritize causal variant was performed using the HyPrColoc package.^28^ Details of analyses are provided in the Supplemental Material.

### Cell Culture

Rat SMCs (A7r5) were purchased from ATCC (Manassas, VA) and maintained in DMEM supplemented with 10% fetal bovine serum (Thermo Fisher Scientific, Waltham, MA). Human iPSC line SKiPS-31.3 was obtained by reprogramming of human dermal fibroblast of a healthy male adult volunteer as previously described.^29^ iPSC line 11.10 was purchased from Cell Applications (San Diego, CA). All iPSCs were maintained in mTeSR Plus medium (STEMCELL Technologies). iPSCs were differentiated in 24 days into mesoderm-derived vascular SMCs as previously described and detailed in the Supplemental Material.^30,31^ After day 24, iPSC-SMCs were maintained in DMEM supplemented with 5-µg/mL insulin, 0.5-ng/mL epidermal growth factor, 2-ng/mL basic fibroblast growth factor, 5% fetal bovine serum, and 1% penicillin-streptomycin (Thermo Fisher Scientific).

### Genome Editing in iPSCs by CRISPR/Cas9

Guide RNAs were designed using the CRISPOR webserver.^32^ Cas9 plasmids pSpCas9(BB)-2A-Puro/pSpCas9(BB)-2A-Hygro, in which we included single guide RNAs, were gifts from Feng Zhang and Ken-Ichi Takemaru (Addgene #62988/#127763).^33^ Details of iPSC transfection and colony isolation are provided in the Supplemental Material.

### Cellular Assays

Cellular assays including chromatin immunoprecipitation, cell viability assay, EdU (5-ethynyl-2´-deoxyuridine) flow cytometry assay, wound healing assay, collagen matrix contraction assay, and intracellular calcium measurement were performed as previously described.^34,35^ Representative images or figures were selected to accurately reflect the average results observed across multiple experiments, ensuring consistency with the overall findings. Details for each method are given in the Supplemental Material.

### Statistical Analyses

Statistical assessment of RNA-Seq was performed using DESeq2 (v1.32.0) package in R (v4.1.0).^36,37^ Differential detection analysis in mass spectrometry experiments was done using DEqMS package (v1.10.0).^38–41^ Unless otherwise noted, statistical significance between independent samples was evaluated using the Wilcoxon rank-sum test. For quantitative PCR (qPCR) experiments involving technical replicates, we used a linear mixed-effect model with lme4 (v1.1-35.5) R package. Further details are given in the Supplemental Material.

### Source Data

See the Major Resources Table in the Supplemental Material.

## Results

### *LRP1* Is a Pleiotropic Locus Involved in the Risk of Multiple Arterial Diseases

**Figure 1.**
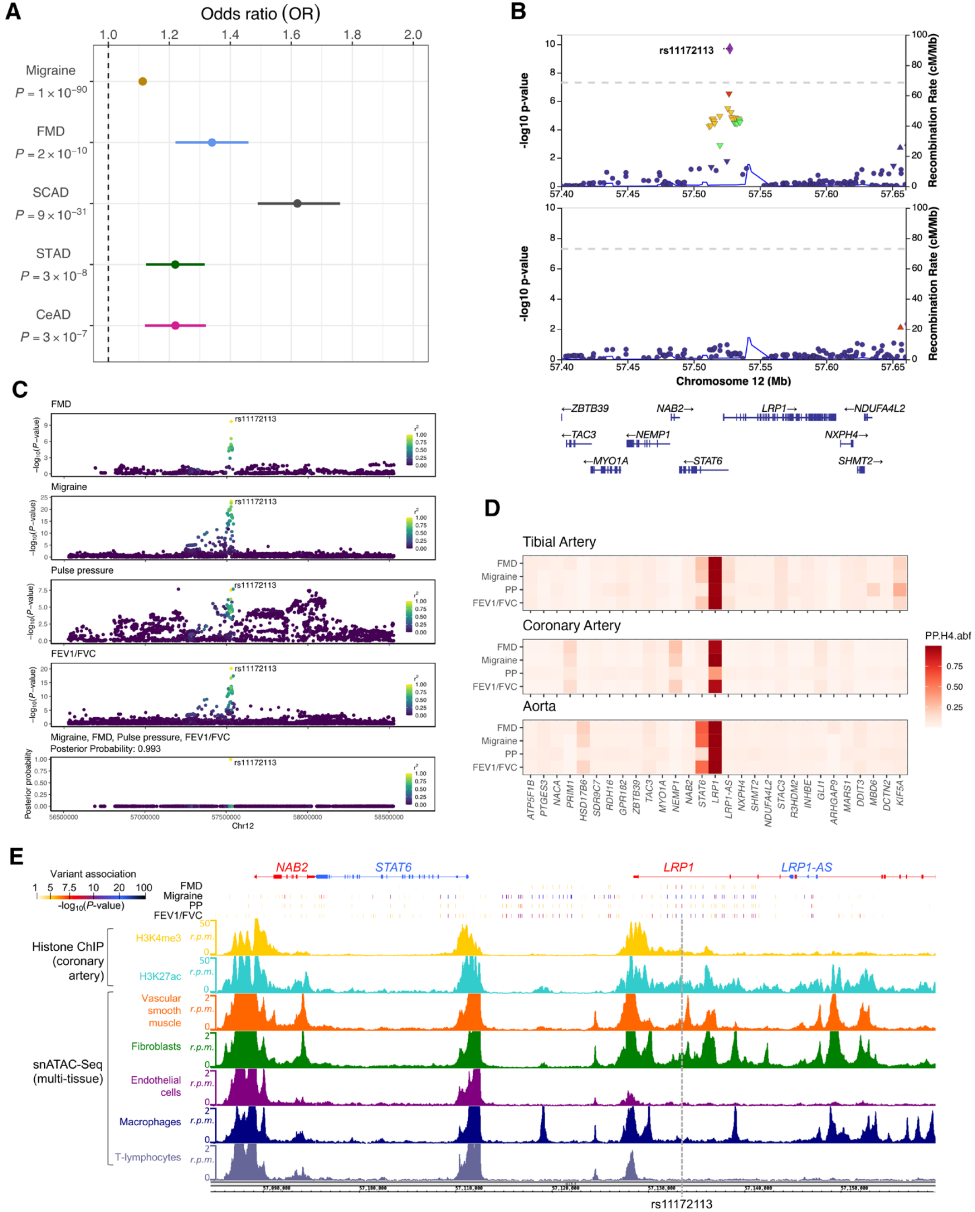
**Genetic associations and functional annotations at the *LRP1* locus. A**, Forest plot representing odds ratio (OR) estimations and *P* values of the associations between rs11172113-T and migraine, fibromuscular dysplasia (FMD), spontaneous coronary artery dissection (SCAD), sporadic thoracic aorta dissection (STAD), and cervical artery dissection (CeAD). **B**, Representative locusZoom plots of FMD association before (**top**) and after (**bottom**) conditioning on rs11172113 association using the GCTA-COJO software.^26^ **C**, Multitrait colocalization analysis performed using the HyPrColoc method.^28^ Genetic association signals for migraine, FMD, and forced expiratory volume over vital capacity (FEV1/FVC) are represented in a 2-Mb region centered on rs11172113. The Association *P* value (log10-scale) of each genetic variant is represented on the *y* axis, while the dot color represents r^2^ of linkage disequilibrium with rs11172113 (1000G-CEU reference panel). **Lower**, The relative posterior probability for each genetic variant to be causal. The total posterior probability for traits to colocalize is given over the graph (posterior probability [PP], 0.993). **D**, Heatmap representing colocalizations of association signals for 4 traits or diseases and expression quantitative trait locus (eQTL) association signals in artery tissues. Genes overlapping a 1-Mb window centered on rs11172113 were tested for colocalization. Tile color represents the H4 coefficient of approximate Bayes factor colocalization (PP.H4.abf, 0–1). **E**, Genome browser visualization of histone chromatin immunoprecipitation (histone-ChIP) and single-nucleus assay for transposase accessible chromatin with high throughput sequencing (ATAC-seq) read densities in reads per million (r.p.m) in the regions surrounding rs11172113. The dashed gray line highlights rs11172113 exact position.

*LRP1* locus was previously reported to be associated with migraine^42^ (odds ratio [OR], 1.11; *P*=1×10^−90^), FMD^11^ (OR, 1.33; *P*=2x10^-10^), SCAD^43^ (OR, 1.62; *P*=9×10^−31^), sporadic thoracic aortic dissection^10^ (OR, 1.21; *P*=3×10^−8^), and suggestively to cervical artery dissection^44^ (OR, 1.22; *P*=3×10^−7^), as summarized in Figure 1A. We applied conditional regression analyses using the GCTA-COJO software^26^ and confirmed a single genetic signal that involves rs11172113 as the lead variant in FMD and migraine signals, for which we had access to full summary statistics (Figure 1B; Figure S1). Multitrait Bayesian colocalization analysis^28^ of association signals for migraine, FMD, PP, and forced expiratory volume over vital capacity indicated high posterior probabilities for colocalization with a single shared causal variant (posterior probability of 99.3% for 4 traits; Figure 1C), supporting rs11172113 to likely explain the observed association signals (Figure 1C). In the same line, colocalization analyses between GWAS and expression quantitative trait locus (eQTL) signals in the aorta, tibial, and coronary arteries from Genotype-Tissue Expression also supported rs111712113 as the likely causal variant in this locus in these tissues, with *LRP1* as the most likely and only target gene (PP.H4.abf>80%; Figure 1D; Figure S2). Of note, no eQTL association signal was observed for rs11172113 in pulmonary tissues although a suggestive eQTL signal, colocalizing with arterial eQTL, is found in fibroblasts (Figure S2).

Altogether, our results support rs11172113 as the most likely causal variant and candidate functional variant potentially involved in *LRP1* expression in arteries.

### rs11172113 Is Located in an Enhancer Region Active in iPSC-Derived SMCs

**Figure 2.**
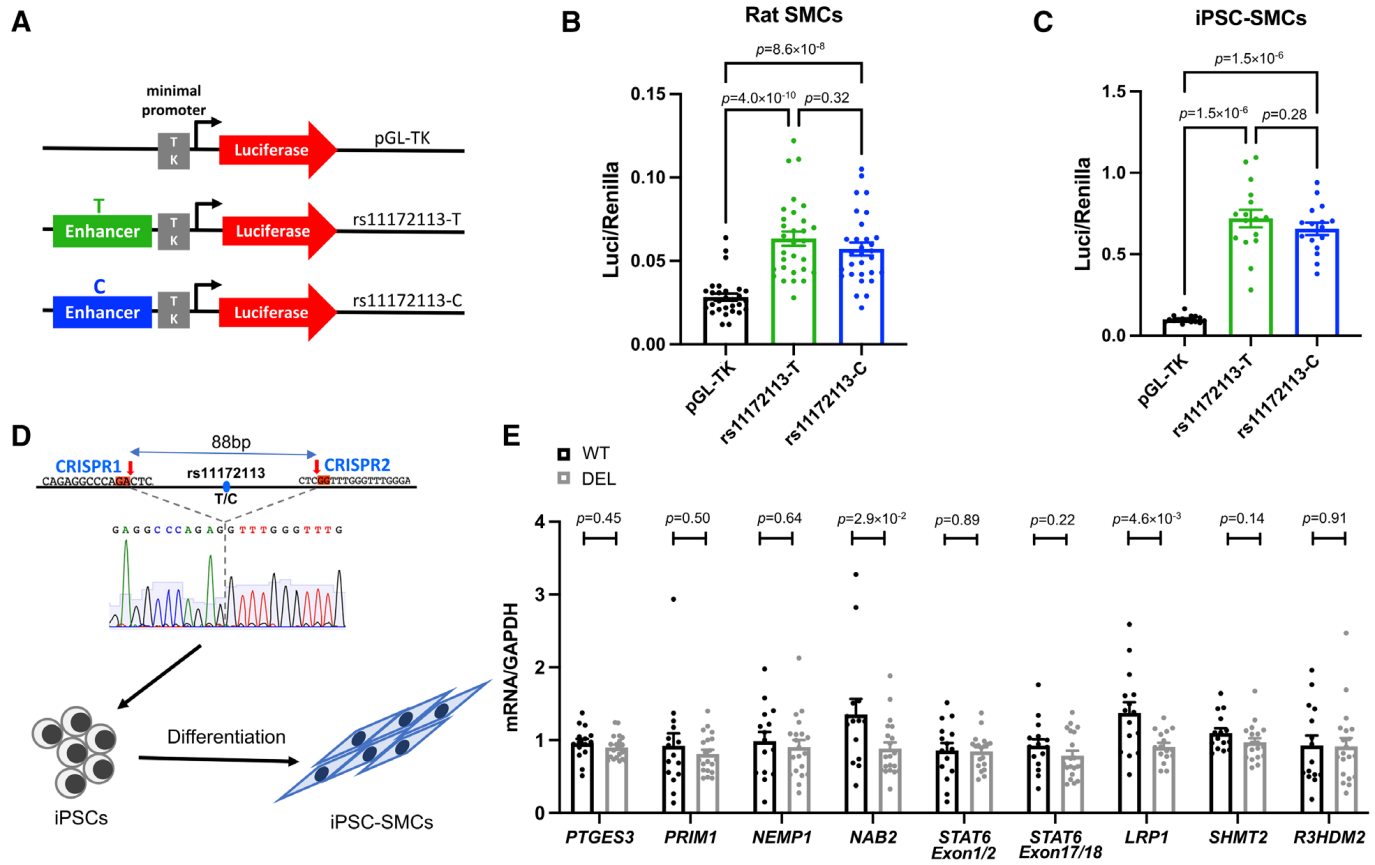
**Enhancer capacity of genomic region harboring rs11172113. A**, Illustration of reporter plasmids including 1-kb rs11172113-centered region for each allele (T/C) inserted into the pGL-TK (plasmid with Gaussia Luciferase and minimal Thymidine Kinase promoter) luciferase plasmid. **B** and **C**, Dual-luciferase assay using empty vectors (gray) and 1-kb region with rs11172113-T (green) or C allele (blue) in rat (**B**; n=30) or induced pluripotent stem cell (iPSC)-smooth muscle cells (SMCs; **C**; n=8). Unadjusted *P* values obtained Wilcoxon rank-sum test are indicated. **D**, Illustration of the region deleted in iPSCs and then differentiated into SMCs (iPSC-SMCs). **E**, Genes relative expressions near *LRP1* locus in iPSC-SMCs with or without rs11172113-associated enhancer. Four wild-type (WT) and 5 enhancer region deletion cell (DEL) clones were analyzed in 2 independent experiments, each assayed twice. Unadjusted *P* values of comparison between WT and DEL samples are indicated (linear mixed model).

We have previously shown that rs11172113 is located within an open chromatin region in primary arterial SMCs and dermal fibroblasts.^11^ Using open chromatin maps generated by single-nucleus assay for transposase accessible chromatin with high throughput sequencing from multiple tissues,^45^ we confirmed rs11172113 to overlap with open chromatin regions specifically in vascular SMCs and fibroblasts but not in endothelial cells, macrophages, or T lymphocytes (Figure 1E). To assay the enhancer activity of this genomic region, we cloned a 1-kb genomic region centered on rs11172113 (T or C allele) in a reporter plasmid (Figure 2A). Enhancer dual-luciferase reporter assays indicated that this sequence produced significantly higher luciferase activity compared with a control sequence, both in rats (Figure 2B) and iPSC-derived SMCs (Figure 2C). However, we observed no significant differences in luciferase activities between T and C allele constructs (Figure 2B and 2C).

To further confirm the enhancer activity of rs11172113 surrounding region, we generated iPSC where we deleted using the CRISPR-Cas9 (Clustered Regularly Interspaced Short Palindromic Repeats-CRISPR associated protein 9) system an 88 bp sequence centered on rs11172113 (Figure 2D). We obtained 5 single clones with sequence deletions (enhancer region deletion cell [DEL]) from 1 female donor iPSC line (11.10 cell line), which we compared with 5 single clones in total without deletion generated using Cas9 overexpression in the absence of guide RNA (Table S1). Compared with their parental cell line, all clones presented similar expressions of pluripotency markers (Figure S3). We differentiated all clones into SMCs throughout the mesodermal lineage as previously described.^30^ All clones but one, which was discarded, had a positive expression of SMC markers *ACTA2* and *TAGLN* (Figure S3B). We found a significant decrease in *LRP1* expression in DEL iPSC-SMCs compared with wild-type (WT) SMCs, supporting an enhancer effect of this region on the expression of *LRP1* (Figure 2E). We also examined the expression of genes mapping in the vicinity of *LRP1* (within ±300 -kb reported to be expressed in arteries). In addition to *LRP1*, we found a significant decrease in the expression of the *NAB2*, located 40 kb upstream of rs11172113 (Figure 2E). rs11172113 is reported in Genotype-Tissue Expression as a splicing QTL in the tibial artery with allele T associated with a lower inclusion of canonical exon 1-exon 2 junction within the transcript for *STAT6* (Figure S4). However, we did not observe changes in the expression of *STAT6* when we targeted exon 1-exon 2 and exon 17-exon 18 junctions (Figure 2E). This result does not support rs11172113 variation as the causal variant for this sQTL described in Genotype-Tissue Expression.

To further replicate these findings in a different genetic background, we generated additional WT and DEL clones in a different iPSC line of male sex (Table S1; 31.3 cell line).^29^ We differentiated all clones into SMCs and verified the expression of SMC markers: smooth muscle actin, smooth muscle myosin heavy chain, transgelin, and calponin 1 using qPCR and Western Blot (Figure S5). We confirmed that *LRP1* expression was reduced by about 20% at both mRNA level and protein level in DEL iPSC-SMCs (Figures S5 and S6) although we detected significant change only at the mRNA level (Figure S6). The expression of *NAB2* was below the detection range in qPCR experiments. Altogether, our results support *LRP1* as the main transcriptional target of the enhancer associated with rs11172113 in vascular SMCs.

### Transcription Factors MECP2 and SNAIL Are Repressors of *LRP1* Expression

**Figure 3.**
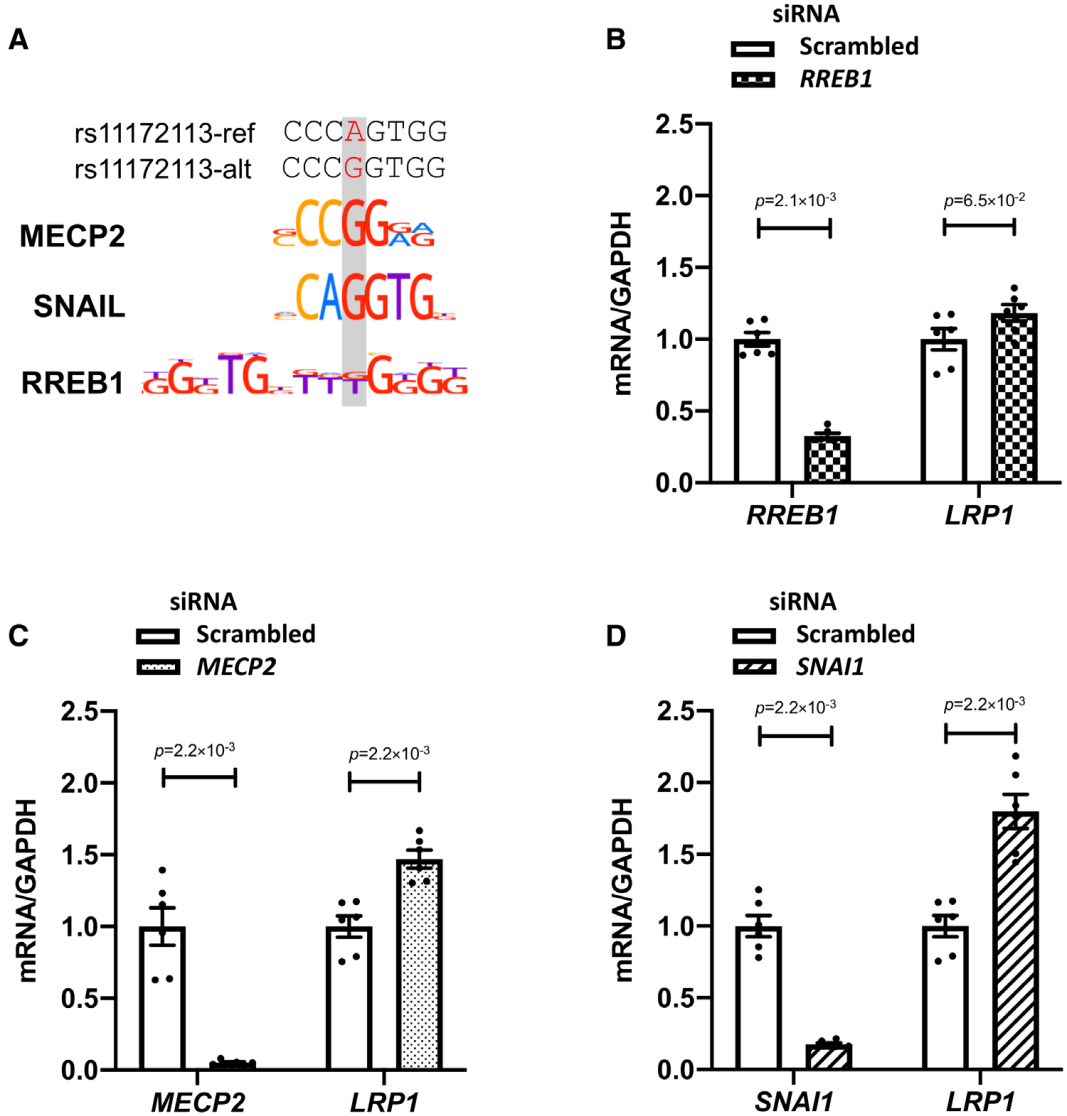
**Transcription factors MECP2 (methyl CpG binding protein 2) and SNAIL (Zinc Finger Protein SNAI1) are repressors of *LRP1* (low-density lipoprotein receptor-related protein 1) expression. A**, Representative images showing the alignment of MECP2, SNAIL, and RREB1 (RAS-responsive element binding protein 1) transcription factor motifs to rs11172113 T or C allele. **B** through **D**, Bar plots showing mean±SEM of relative RNA levels of *RREB1* (**B**), *MECP2* (**C**), *SNAI1* (**D**), and *LRP1* detected using quantitative PCR following treatment with siRNAs targeting *RREB1* (**B**), *MECP2* (**C**), and *SNAI1* (**D**) compared with scrambled siRNA. Unadjusted *P* values from the Wilcoxon rank-sum test are indicated (n=6).

We used PERFECTOS-APE software^46^ to assess potential changes in the binding capacity or recruitment of TFs (transcription factors) according to rs11172113 alleles. We found 10 candidate TFs predicted to recognize differentially the sequence including rs11172113 (log2-fold change >2; Table S2). Among these, 7 TFs were reported to be expressed in artery tissues from Genotype-Tissue Expression. We prioritized 3 TFs that had functions relevant to arterial disease (Figure 3A): the methyl CpG binding protein 2 (MECP2) that exhibits a regulatory role in VSMC phenotypic modulation and neointima formation^47,48^; *SNAI1* that encodes zinc finger protein SNAIL, a member of a family of transcriptional repressors involved in the control of endothelial and epithelial to mesenchymal transitions and fibroblast activation^49,50^; and RREB1 (RAS-responsive element binding protein 1), an RAS transcriptional effector known to cooperate with TGF-β/SMAD (Mothers against decapentaplegic homolog) pathway to regulate epithelial to mesenchymal transition.^51^ All 3 TFs were abundantly expressed in SMCs, fibroblasts, and endothelial clusters inquired from a single-nucleus RNA-Seq dataset of diseased human coronary arteries,^52,53^ with *MECP2* showing higher expression in the SMC cluster, compared with *SNAI1* and *RREB1* (Figure S7).

**Figure 4.**
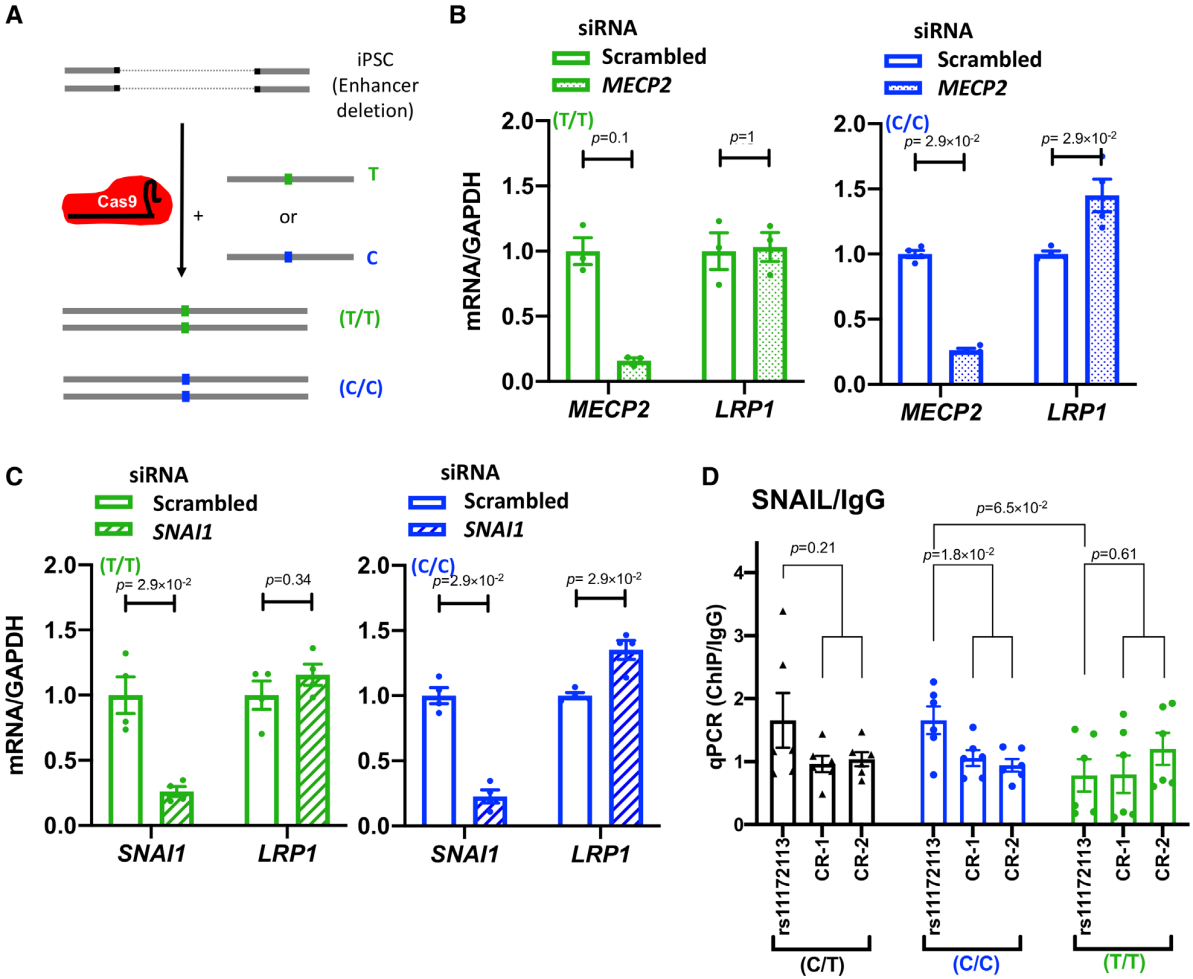
**SNAIL (Zinc Finger Protein SNAI1) selectively binds to the rs11172113-C allele. A**, Illustration of cloning strategy to generate induced pluripotent stem cells (iPSCs) with rs11172113 homozygous alleles. **B** and **C**, Bar plots showing mean±SEM of relative RNA levels of *MECP2* (**B**), *SNAI1* (**C**), and *LRP1* (low-density lipoprotein receptor-related protein 1) detected using quantitative PCR (qPCR) following treatment with siRNAs targeting *MECP2* (**B**; n=3) and *SNAI1* (**C**; n=4) compared with scrambled siRNA in iPSC-smooth muscle cells (SMCs) harboring homozygous rs11172113-TT (green; **left**) or rs11172113-CC (blue; **right**) genotypes. **D**, Chromatin immunoprecipitation (ChIP) targeting SNAIL in iPSC-11.10 and homozygous rs11172113-CC or rs11172113-TT (2 clones each). Immunoprecipitated material was evaluated using qPCR targeting and rs11172113 region and 2 control regions (no enhancer marks in SMCs or artery tissue). The ratio of SNAIL ChIP to ChIP with rabbit IgG is given. Each clone was assessed in 3 independent experiments. Unadjusted *P* values from the Wilcoxon rank-sum test are indicated.

We found a significant increase in *LRP1* expression when iPSC-derived SMCs (CT genotype for rs11172113) were treated with siRNA targeting *MECP2* and *SNAI1* but not *RREB1* (Figure 3B and 3D), supporting a repression of *LRP1* expression by MECP2 and SNAIL. To determine whether this repression was dependent on rs11172113, we generated homozygous iPSCs for each allele (Figure 4A; Figure S8; Table S1). SMCs derived from each genotype presented positive expression of SMC markers but no significant difference in the expression levels of *LRP1* (Figure S9). However, the knockdown of *MECP2* or *SNAI1* resulted in a significant increase of *LRP1* expression only in iPSC-SMCs with the C/C genotype (Figure 4B and 4C). Chromatin immunoprecipitation using antibodies for both TFs in iPSC-SMCs with each genotype (C/C, C/T, or T/T) confirmed SNAIL (Figure 4D) but not MECP2 (Figure S10) to bind to this enhancer only in cells with the C/C genotype (Figure 4B and 4C).

In summary, these data suggest that the rs11172113-C allele is specifically recognized by SNAIL, a repressor of transcription expressed in VSMCs.

### *LRP1* Knockout Induces TGF-β Activation and ECM Remodeling in iPSC-SMCs

**Figure 5.**
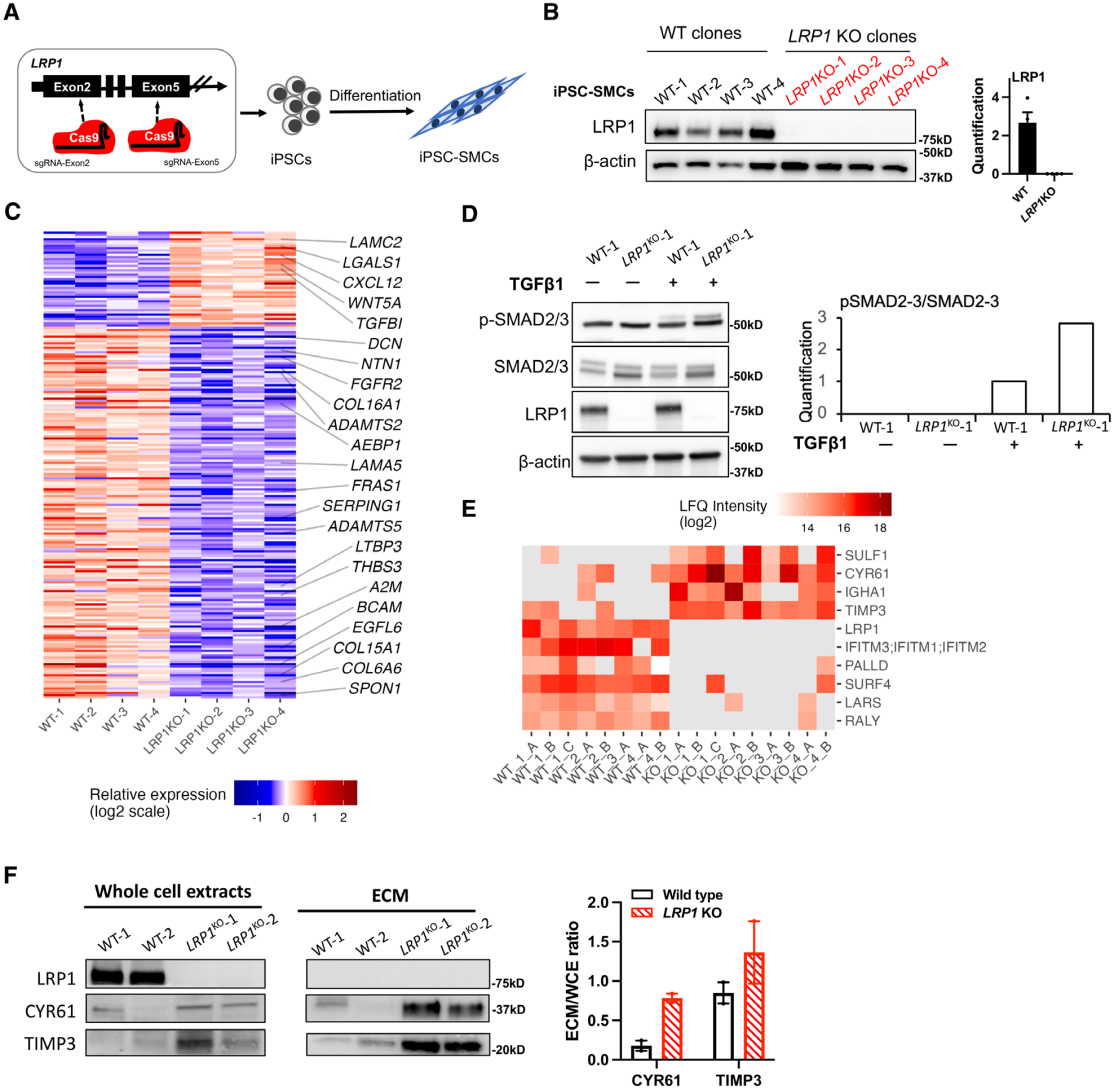
**Transcriptomic and proteomic changes following *LRP1* (low-density lipoprotein receptor-related protein 1) knockout (KO) in induced pluripotent stem cell (iPSC)-smooth muscle cells (SMCs). A**, Illustration of gRNA position to generate *LRP1* KO iPSCs. **B**, Representative Western blot images and quantification showing LRP1 protein expression in wild-type (WT) and *LRP1* KO iPSC-SMCs. **C**, Heatmap representation of relative RNA expression for differentially expressed genes between *LRP1* KO and WT iPSC-SMCs. Genes involved in the Gene Ontology biological process labeled (collagen-containing extracellular matrix) are indicated. **D**, Representative Western blot images and quantification showing the expression of p-SMAD2/3 (phosphorylated Mothers against decapentaplegic homolog 2/3) and total SMAD2/3 (SMAD2/3) in WT and *LRP1* KO iPSC-SMCs in the presence of TGFβ (transforming growth factor beta) 1 protein for 1 hour. **E**, Heatmap representation of label-free quantification (LFQ) for proteins over or underrepresented in extracellular extracts of *LRP1* KO and WT iPSC-SMCs. **F**, Representative Western blot images showing the expression of LRP1, CYR61 (Cystein rich angiogenic protein 61), and TIMP3 (Metalloproteinase inhibitor 3) in whole cell extracts (WCEs) or decellularized extracts (extracellular matrixes [ECMs]) and the quantification ratio of ECM to WCE.

Given the existing conflicting data on LRP1's role in SMCs, we aimed to assess the cellular consequences of LRP1 loss of function in a human iPSC-SMC model. We introduced a frameshift indel insertion at either exon 2 or exon 5 of *LRP1* using CRISPR/Cas9 (Figure 5A) and obtained 4 *LRP1* knockout iPSC clones with a complete loss of LRP1 protein expression (Figures S11 and S12; Table S1), including after differentiation into SMCs (Figure 5B; Figure S11). Compared with WT, we found a decrease in proliferation and migration capacities in *LRP1* knockout iPSC-SMCs (Figures S13 through S16). Data in mice described LRP1 as a ligand for intracellular calcium channels in SMCs, promoting their contractile function.^21^ However, the ability of our *LRP1* knockout iPSC-SMC model to contract a collagen gel lattice or on intracellular calcium release in response to 2 contractile inducers (carbachol and angiotensin II) was not significantly different from WT iPSC-SMCs (Figures S17 and S18).

Global gene expression changes using bulk RNA sequencing indicated 213 genes to be differentially expressed between WT and *LRP1* knockout cells, with a majority of genes upregulated in the *LRP1* knockout cells (Figure 5C). Differentially expressed genes were enriched for genes involved in collagen-containing ECM (*P*=3.1×10^−11^; Figure 5C; Figure S19; Table S3), connective tissue development (*P*=3.2×10^−6^), and lung development (*P*=3.3×10^−6^; Figure S19; Table S3). Among genes deregulated in *LRP1* knockout, we highlight the *TGFBI*, an exocrine protein induced by the TGF-β signaling pathway^54^ (Figure 5C). Interestingly, we found an increased amount of p-SMAD2/3 in *LRP1* knockout SMCs after stimulation by TGF-β1 for 1 hour and no change in total SMAD2/3 (Figure 5D), confirming that canonical TGF-β signaling is enhanced in this model.

The regulation of ECM deposition was the main impaired pathway in our *LRP1* knockout iPSC-SMCs, a key pathway in several arterial diseases that are specifically suspected in the case of FMD and SCAD.^55,56^ To investigate further this mechanism, we assayed changes using label-free quantification with MaxQuant software^40^ of ECM composition by mass spectrometry of decellularized extracts of *LRP1* knockout and WT iPSC-SMCs. Despite the similar protein content of most abundant ECM proteins (Figure S20), several proteins were systematically detected only in 1 of the 2 conditions. In particular, *LRP1* knockout SMCs secreted increased levels of CYR61, a cysteine-rich angiogenesis inducer, and a known ligand for LRP1 involved in apoptosis, adhesion, migration, and vascular integrity.^57^ We also found higher amounts of TIMP3, an inhibitor of the matrix metalloproteinases involved in ECM degradation and known to bind the extracellular domain of LRP1 to facilitate the clearance of target metalloproteinases (Figure 5E). *CYR61* and *TIMP3* were confirmed to be abundantly expressed in SMC, fibroblasts, and endothelial clusters inquired from single-nucleus RNA-Seq dataset of diseased human coronary arteries^52^ (Figure S7). Immunoblotting on whole cell or ECM extracts generated from iPSC-SMCs confirmed the presence of increased amounts of CYR61 and TIMP3 in *LRP1* knockout SMCs compared with WT cells (Figure 5E), which we confirmed in iPSC-SMCs after LRP1 knockdown for 3 days (Figure S21).

### Deletion of rs11172113-Associated Enhancer Potentiates TGF-β Signaling

**Figure 6.**
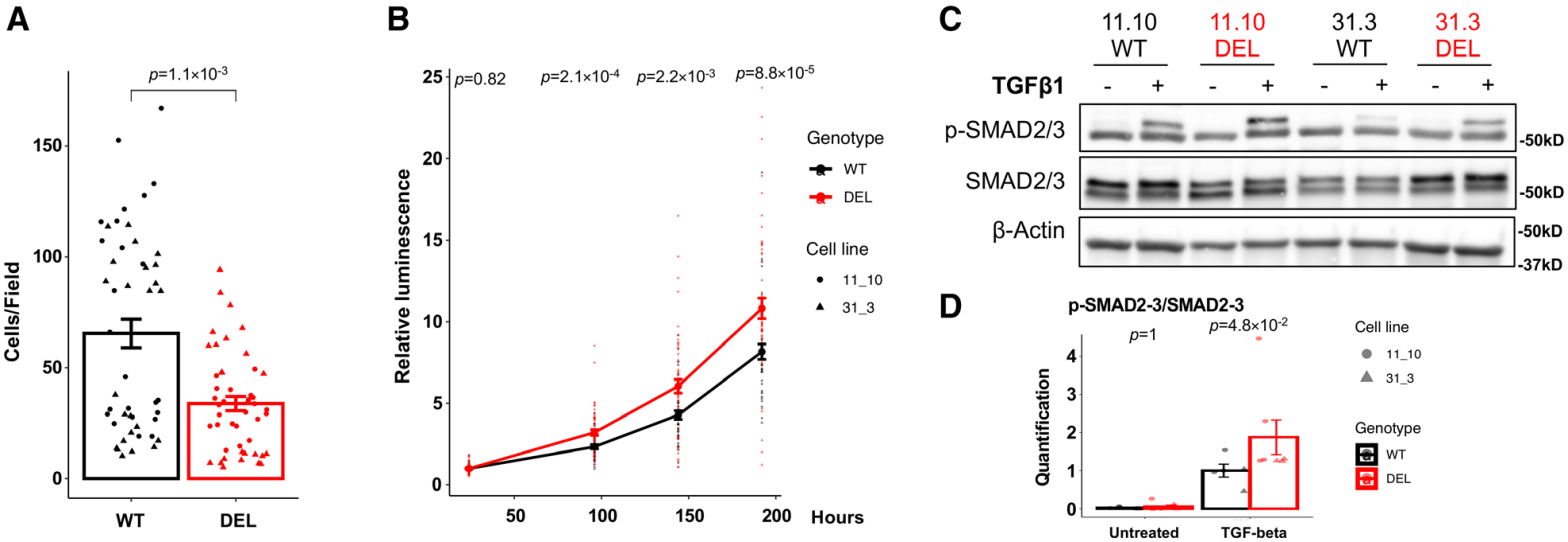
**Cellular phenotypes of induced pluripotent stem cell (iPSC)-smooth muscle cells (SMCs) with and without rs11172113 associated enhancer deletion. A**, Transwell cell migration assay in wild type (WT; black dots; n=24 from 4 clones) and rs11172113-associated enhancer region deletion cell (DEL) SMCs (gray squares; n=24 from 4 clones). Barplot representing mean±SEM of the number of cells counted in each field following transwell assay assessing cell migration through a polycarbonate membrane. **B**, Cell proliferation in iPSC-SMCs. Measurement of cell viability (mean±SEM) of WT (black dots; n=40 from 5 clones) and DEL SMCs (gray squares; n=48 from 6 clones) at 24, 96, 144, and 192 hours after the end of differentiation. **C**, Representative Western blot images showing the expression of p-SMAD2/3 (phosphorylated Mothers against decapentaplegic homolog 2/3) and total SMAD2/3 (SMAD2/3) in WT and DEL iPSC-SMCs in the presence of TGFβ (transforming growth factor beta) 1 protein for 1 hour. **D**, Quantification based on Western blot images from **C** and Figure S22 (5 WT clones and 7 DEL clones). Barplot represents the mean±SEM of p-SMAD2/3 over SMAD2/3 ratio. Unadjusted *P* values from the Wilcoxon rank-sum test are indicated.

Given the altered cellular function of *LRP1* deficiency in the iPSC-SMC model, we aimed to investigate the functional impact of rs11172113 associated enhancer on the iPSC-SMC model. Compared with WT cells, we found a decrease in the migration capacity of iPSC-SMCs with DELs, which is consistent with the observations in *LRP1* knockout SMCs (Figure 6A). Conversely, we found a slightly but significantly increased proliferation capacity in DEL iPSC-SMCs (Figure 6B). We then tested the activation of canonical TGF-β signaling by treating starved iPSC-SMCs with TGF-β1 for 1 hour. Interestingly, we found an increased ratio of p-SMAD2/3 to total SMAD2/3 in DEL iPSC-SMCs (Figure 6C and 6D; Figure S22), similar to the observation in *LRP1* knockout iPSC-SMCs.

To identify differential changes in gene expression, which could mediate these phenotypic differences, we analyzed mRNA expression in WT and DEL iPSC-SMCs using bulk RNA sequencing. We found 5 differentially expressed genes of unclear relevance, suggesting that the observed phenotypic changes might be due to direct LRP1 effect rather than transcriptomic changes (Figure S23). Focusing on nominal differences in gene expression (unadjusted *P*<0.05), we, however, found an enrichment of genes involved in ECM organization (*P*=1.1×10^−6^) and extracellular structure organization (*P*=4.3×10^−6^) among differentially expressed genes (Figure S23; Table S4), consistent with findings in *LRP1* knockout cells. We, however, detected no changes in the amounts of ECM proteins, CYR61 and TIMP3, secreted by DEL iPSC-SMCs compared with WT cells (Figure S24).

Collectively, these data indicate that the enhancer deletion–induced reduction in *LRP1* expression partially captures the altered SMC function induced by complete *LRP1* deficiency.

## Discussion

In this integrated study, we provide molecular and cellular mechanisms underlying the genetic association in the *LRP1* locus involved in the risk for multiple major vascular diseases. Through conditional analyses and colocalization of association signals, we pointed at the intronic variant rs11172113 as the most likely causal variant for all associations reported. In vitro experiments conducted on several genome-edited iPSC–derived SMC models support rs11172113 to be located in an enhancer region that controls the expression of *LRP1* through the action of transcription factors MECP2 and SNAIL in an allele-specific repression mechanism. Our results also support *LRP1* depletion to decrease iPSC-derived SMC proliferation and migration, potentiate canonical TGF-β signaling, and lead to partial ECM remodeling involving enhanced secretion of CYR61 and TIMP3 proteins. We found that enhancer deletion–induced reduction in *LRP1* expression partially captures the altered SMC function induced by complete *LRP1* deficiency.

Our results illustrate the complexity of regulatory mechanisms at stake at GWAS loci involving potentially multiple target genes. Following the deletion of an 88bp region centered on the most probable causal variant in this locus in iPSC-derived SMCs, we found a significant and consistent decrease in the expression of *LRP1*. Although we cannot exclude the possibility of >1 target gene in this locus, which may explain its high genetic pleiotropy, genetic colocalization of eQTL signals in arterial tissues with GWAS signals supported *LRP1* as the most likely causal gene at the locus. Future studies involving potentially additional cell types, relevant to vascular injury, for instance, may, however, lead to additional relevant findings.

Using in silico prediction and gene knockdown in genome-edited cells, we found that transcription factors, SNAIL and MECP2, repress *LRP1* expression specifically in the presence of the C allele of rs11172113. Through chromatin immunoprecipitation experiments, we confirmed in silico predictions and showed that SNAIL directly binds to the rs11172113 region in an allelic-specific manner. SNAIL is a master regulator of endothelial-to-mesenchymal transition involved in tumor metastasis, kidney fibrosis, and pulmonary hypertension.^58–60^ Although the function of SNAIL in vascular SMCs is not known, it was recently found that closely related transcription factor SLUG was involved in SMC phenotypic regulation during atherosclerosis.^61^ The absence of interaction with MECP2 could be due to technical limitations of the chromatin immunoprecipitation approach, in particular, the limited availability of chromatin immunoprecipitation grade antibodies. Our result does not rule out a potential role of this TF in *LRP1* regulation, as it was previously shown to regulate vascular SMC phenotypic plasticity and neointima formation.^47,48^

Our findings suggest that the functional consequence of rs11172113 allelic variation could be at play when vascular SMCs and fibroblasts undergo major phenotypic changes, such as wound repair, neointima formation, arterial remodeling, and atherosclerosis, as reported in several in vivo studies.^19,21,22,62^ Conversely, a lack of *LRP1* repression following arterial injury would be expected to impair arterial contractility and wound repair, 2 suspected mechanisms in FMD and migraine pathogenesis.^11,63^ Of note, we did not observe rs11172113 allele-specific differences in *LRP1* expression between iPSC-derived SMCs in the absence of TF knockdown. Potential compensatory mechanisms may buffer the expression of *LRP1*, especially in the absence of the phenotypic transitions that cannot be modeled in our in vitro modeling, which may potentially involve additional TFs with affinity to this enhancer region.

LRP1 is a multifunctional protein that recognizes hundreds of ligands and functions in a variety of biological processes, including lipid metabolism, endocytosis, and signaling pathway regulation.^18,64^ Due to this versatility, the phenotypic effect of LRP1 deletion is highly model-dependent, and limited extrapolation is warranted from in vitro studies. However, through an exploration of molecular changes in *LRP1* knockout iPSC-SMCs, we observed several alterations particularly relevant to the pathogenesis of FMD, arterial dissection, migraine, and lung function. We found that LRP1 deficiency led to an alteration of the ECM both in terms of gene transcription and protein content, a finding consistent with a recent study in mice.^65^ The hyperactivation of canonical TGF-β signaling in LRP1 deficient cells may explain changes in gene expression, given the role of this pathway in promoting fibrosis provided from in vivo models.^62,66^ In support of this mechanism, we mention the enrichment for rare mutations in genes from the TGF-β pathway among patients with SCAD,^67^ the recent report of *FBN1*, a known reservoir for TGF-β, as a potential target gene in a SCAD risk locus,^12,68^ and *TGFBR2* in migraine.^69^ Thus, increased expression of *LRP1* in arteries, as genetically predicted in SCAD, FMD, and migraine in *LRP1* allele risk carriers, could preclude the dysregulation of TGF-β signaling.

Our study also identified an increased deposition of TIMP3 and CYR61 in the ECM generated by LRP1 knockout SMCs. TIMP3 promotes the degradation and clearance of matrix metalloproteinases and is suspected to be involved in a wide variety of cardiovascular diseases.^70^ LRP1 regulation of TIMP3 endocytosis is well documented in a variety of models.^70^ In addition, *TIMP3* was recently prioritized in a SCAD GWAS, which supports a potential mechanistic link between LRP1 and TIMP3 in the context of this arterial disease.^68^ On the other hand, CYR61 is a paralogue of connective tissue growth factor (CTGF), reported to be regulated by *LRP1* in arteries.^20,62^ CYR61 plays an important role in wound healing and was associated with several lung diseases, including chronic obstructive pulmonary disease and pulmonary hypertension,^71,72^ consistent with *LRP1* association with FEV/FEC. However, the relevance of this mechanism in other vascular diseases needs further investigation.

Our work presents several limitations. First, iPSC-derived SMCs are a useful model but do not fully recapitulate all complex phenotypes of SMCs in vivo, which warrants further confirmation of the proposed regulatory mechanisms. Second, we did not prioritize the investigation of the function of *NAB2* in SMCs based on nonconsistency between qPCR findings and the existing eQTLs data from human arteries compared with *LRP1*. Thus, we cannot exclude the role of this gene in the physiopathology of vascular diseases with the genetic association reported in this locus. Third, we did not investigate, in detail, the phenotypic and transcriptomic changes linked to the rs11172113 genotype only. The effect size of the eQTL association observed in arteries limited the experimental design involving a reasonable number of clones to model this effect in vitro. Thus, our alternative approach to analyze the function of rs11172113 in the context of the associated enhancer may only partially capture the specific impact of the rs11172113 genetic effect. Fourth, although we showed that TFs, MECP2 and SNAIL, regulate the expression of *LRP1* specifically through rs11172113 C allele, we did not demonstrate direct binding of MECP2 to rs11172113 associated enhancer, and additional TFs predicted in silico to change their ability to bind to this genomic enhancer may also play a regulatory role. Finally, our study design does not cover the highly suspected multiple cell type interactions involving endothelial cells and fibroblasts to fully depict the functional translation of the genetic association with such a large panel of arterial diseases.

In conclusion, we provide a comprehensive functional investigation of a highly pleiotropic genetic locus involved in the genetic susceptibility to several vascular diseases and propose plausible molecular and cellular mechanisms linking the most likely causal variant to gene expression regulation of *LRP1*. We provide evidence for an important impact of the most likely target gene, *LRP1*, on the regulation of proliferation and migration. Based on the exploration of the ECM synthesis capacity of several in vitro iPSC-SMC models, we propose a mechanism involving, at least partially, potentiated activity of the canonical TGF-β signaling. Our work provides genetically driven biological mechanisms to elucidate the pathogenesis of several important and understudied cardiovascular diseases.

## ARTICLE INFORMATION

### Acknowledgments

This work benefited from expertise and support from the induced pluripotent stem cells Technical Facility at Paris Cardiovascular Research Center (PARCC) and the Institute for Integrated Cell Biology (I2BC) High Throughput Sequencing Core Facility. The Genotype-Tissue Expression project was supported by the Common Fund of the Office of the Director of the National Institutes of Health and by the National Cancer Institute, the National Human Genome Research Institute, the National Heart, Lung, and Blood Institute, the National Institute on Drug Abuse, the National Institute of Mental Health, and the National Institute of Neurological Disorders and Stroke.

### Sources of Funding

This study was supported by the European Research Council grant (ERC-Stg-ROSALIND-716628 to N. Bouatia-Naji), the French Society of Cardiology through Fondation Coeur et Recherche (to N. Bouatia-Naji), and the Fédération Française de Cardiologie (to N. Bouatia-Naji). L. Liu and Y. Liu were supported by 2 PhD scholarships from the Chinese Scientific Council.

### Disclosures

None.

### Supplemental Material

Expanded Material and Methods

Tables S1–S4

Figures S1–S24

References 26–41

## Supplementary Material
